# Brain Reorganization following Intervention in Children with Congenital Hemiplegia: A Systematic Review

**DOI:** 10.1155/2013/356275

**Published:** 2013-12-03

**Authors:** E. Inguaggiato, G. Sgandurra, S. Perazza, A. Guzzetta, G. Cioni

**Affiliations:** ^1^Scuola Superiore Sant'Anna, Piazza Martiri della Libertà, I-56127 Pisa, Italy; ^2^Department of Developmental Neuroscience, IRCCS Stella Maris Scientific Institute, Via dei Giacinti 2, Calambrone, I-56128 Pisa, Italy; ^3^Physical and Rehabilitation Medicine, University of Rome Tor Vergata, I-00173 Rome, Italy; ^4^Department of Clinical and Experimental Medicine, University of Pisa, I-56126 Pisa, Italy

## Abstract

Noninvasive rehabilitation strategies for children with unilateral cerebral palsy are routinely used to improve hand motor function, activity, and participation. Nevertheless, the studies exploring their effects on brain structure and function are very scarce. Recently, structural neuroplasticity was demonstrated in adult poststroke patients, in response to neurorehabilitation. Our purpose is to review current evidence on the effects of noninvasive intervention strategies on brain structure or function, in children with unilateral cerebral palsy. The main literature databases were searched up to October 2013. We included studies where the effects of upper limb training were evaluated at neurofunctional and/or neurostructural levels. Only seven studies met our selection criteria; selected studies were case series, six using the intervention of the constraint-induced movement therapy (CIMT) and one used virtual reality therapy (VR). CIMT and VR seem to produce measurable neuroplastic changes in sensorimotor cortex associated with enhancement of motor skills in the affected limb. However, the level of evidence is limited, due to methodological weaknesses and small sample sizes of available studies. Well-designed and larger experimental studies, in particular RCTs, are needed to strengthen the generalizability of the findings and to better understand the mechanism of intervention-related brain plasticity in children with brain injury.

## 1. Introduction

Unilateral cerebral palsy (U-CP) is the most common type of cerebral palsy (CP), with an incidence of 1 in 1000 live-births [1]. Typically, the upper limb (UL) is more involved than the lower, with impairments of spasticity, sensation, and reduced strength. Effective use of the arm and hand to reach, grasp, release, and manipulate objects is often compromised. Children with hemiplegia usually have the intellectual capacity to attend regular school; however, impaired arm function restricts their participation in educational, leisure, and later vocational roles [2].

U-CP can result from a wide variety of brain lesions, with respect to the timing of insults (acquired during the pre-, peri- or postnatal period), and the type of structural pathology (brain malformations, periventricular lesions, and corticosubcortical lesions) [3]. U-CP often leads to delays in motor development or deconditioning of the affected limb, as individuals are inclined to functional compensation with the intact limb rather than attempting to use the involved limb [4]; this may result in suppression of development of cortical representation of the affected limb, and it may further inhibit its functional use [5, 6]. When the lesion occurs at an early stage of development, either during the intrauterine life or soon after birth, the mechanisms of plastic (re-)organization of the sensory motor system can be different from those observed at later stages of development [7]. Primary motor control of the hemiplegic upper limb can be eventually maintained within the spared tissue of the affected hemisphere (ipsilesional reorganization), or it can be reorganized within the unaffected hemisphere, as a result of the complete withdraw of the crossing fibers from the affected hemisphere and the survival of the fast-conducting ipsilateral motor projections from the unaffected one (contralesional reorganization) [8]. The type of reorganization can be influenced by the size and site of damage, but it appears strongly influenced also by the experience following damage, that is, by the complex interaction between residual motor output from the affected hemisphere and somatosensory feedback from the affected limb [9].

In general terms, adaptive plasticity of the central nervous system (CNS) refers to functional and structural changes in the brain, which are advantageous to offset or improve functions; the term denotes several capacities including the ability to adapt to changes in the environment and to store information in memory associated with learning [10]. There is abundant evidence that the structure of certain brain circuits can change in response to environmental stimuli [11]. Recently, structural neuroplasticity has been demonstrated in response to neurorehabilitation intervention in adult poststroke patients. Gauthier et al. [12] have shown in stroke patients treated with CIMT a significant increase in gray matter volume in several regions, including bilateral primary sensory and motor areas, both hippocampi, and anterior supplementary motor area contralateral to the motor deficit [12].

In children with U-CP, several types of intervention have been used to improve abilities of the affected limb (e.g., neurodevelopmental treatment, neuromuscular electrical stimulation, constraint-induced movement therapy, etc.). Compared to adult poststroke research, a relatively small number of studies investigated the effects of rehabilitation on brain reorganization. The purpose of this study has been to evaluate current evidence on brain reorganization in children with U-CP following noninvasive intervention strategies.

## 2. Methods

Articles were identified through comprehensive searches of computerized bibliographic databases: PubMed, MedLine (1973 to October 2013), CINAHL (Cumulative Index to Nursing and Allied Health Literature) (up from 1994 to October 2013), Web of Science (1992 to October 2013), and ERIC (pre-1966 to October 2013). We also searched for reviews on this topic on the Cochrane Central Register of Controlled Trials, with no result.

The search explored Medical Subject Headings (MeSH) terms and text words:

1. “cerebral palsy” or “hemiplegia”,
2. “child” or “adolescent” or “infant”,
3. “therapy” or “training" or “intervention”,
4. “MRI” or “fMRI” or “EEG” or “TMS” or “PET” or “MEG” or “reorganization”.

*Selection Criteria.* To be included in this systematic review, studies had to meet the following criteria.

1. Participants were diagnosed with U-CP.
2. Interventions to improve outcome were noninvasive and did not include drugs.
3. Outcomes included functional activities and evidence of brain reorganization through neurophysiological experiments, carried out before and after the intervention.

Studies were excluded if they

1. reported only clinical measures as outcomes;
2. were case reports;
3. were not published in English.

**Figure 1:**
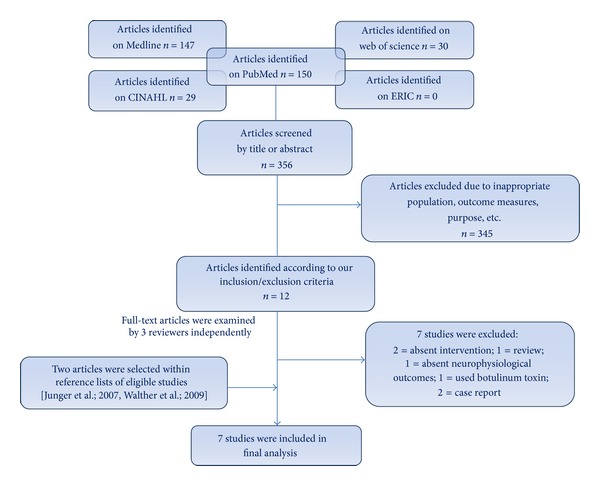
Flow chart of search strategy and selection process.

The initial search yield was reviewed by only one reviewer on the basis of title and abstract. All the studies emerged from the search focused on upper limb (UL) intervention. The search strategy allowed to identify 12 articles that met our inclusion/exclusion criteria. The full-text articles were examined by 3 reviewers, and the eligibility for study inclusion was assessed independently; in case of mismatched opinion between the 3 reviewers, the eligibility of the study was discussed together and consensus was reached. Following our search in the different databases, only 5 eligible studies were identified while two additional ones were selected within their reference lists. The final analysis included 7 studies. The general purpose of the studies was to evaluate the effects of noninvasive rehabilitation strategies on brain reorganization and on functional improvement of affected upper limb (UL) in unilateral cerebral palsy. In Figure 1, flow chart describes study selection and reasons for exclusion.

## 3. Results

### 3.1. Study Designs and Participants

Selected studies were case series; no controlled studies were found.

We found seven trials specifically targeted on children with unilateral cerebral palsy; only in one case a participant had bilateral impairment with right arm sparing [13]. The age range of participants was between 2.1 and 7.6 years in one study [14], between 7 and 14 years in two studies [13, 15], between 13 and 15 years in another study [16], and between 10 and 30 years in the others [17–19]. Some studies were performed by the same research group and some subjects participated in more than one study [17–19].  Table 1 summarizes the characteristics of the population for each study.

### 3.2. Type of Interventions

The most frequently proposed intervention, in six of the seven studies, was the constraint-induced movement therapy (CIMT); CIMT was used in association with neurodevelopmental treatment (NDT) [15], in association with occupational therapy (OT) [13], in association with intensive motor training [14] or during a training camp in association with individual and peer groups activities [17–19]. Standard CIMT for children involves a restraint worn on the non-affected upper limb for 90% of waking hours and 6 hours/day of intensive intervention using shaping techniques and massed practice typically over a 2-week period [20]. The standard CIMT model has been adapted to be less intensive (<6 hours/day) due to concerns that young children are unable to participate in such intensive therapy regimen [21]. The remaining study employed an innovative treatment strategy: virtual reality (VR) [16], a virtual environment system that uses new technologies to make the patient perceptions similar to those coming from real-life activities. In none of the studies children received botulinum injections or upper limb surgery for the affected upper limb in the 6 months prior to intervention.  Table 2 describes the characteristics of each UL intervention.

### 3.3. Outcome Measures

Selected studies were case series; no controlled studies were found. Studies aimed to evaluate the effects of noninvasive intervention on (i) functionality of UL, through scales and/or questionnaires and (ii) brain reorganization, through neuroimaging and neurophysiological techniques (i.e., MRI, fMRI, TMS, MEG). In three of the studies [17–19], the different patterns of corticospinal reorganization (ipsilesional versus contralesional) were determined by using TMS. Outcome measures were applied both before and after the intervention; in a few studies, assessments were also recorded during followup [15, 18].

#### 3.3.1. Upper Limb Function

The effects of noninvasive intervention on the functionality of the hemiplegic upper limb were monitored using different functional measures, scales, or questionnaires. The type of clinical assessments varied among studies; we therefore grouped the functional motor outcomes in 3 categories, according to the dimensions of the International Classification of Functioning. Disability and Health (ICF-CY): (a) body functions and structures, (b) activities, and (c) participation [22]. (Table 1s summarizes clinical assessment and corresponding results; see Table 1s in the Supplementary Material available online at http://dx.doi.org/10.1155/2013/356275). Most of the studies investigated functional motor outcomes according to at least one of the dimensions of the International Classification of Functioning, Disability and Health (ICF). The most frequently used outcomes measures were WMFT [17–19] in 3/7 papers and P-MAL [13, 14, 18] in 3/7 papers.

#### 3.3.2. Brain Reorganization

To evaluate the effects of the intervention on brain reorganization, 6 studies used functional magnetic resonance imaging (fMRI) [13, 15–19]. All MRI experiments were performed on 1.5T scanner, but the fMRI procedure and the tasks performed during the examination were different among studies. Half of the six studies used, as fMRI task, active and passive movements of the paretic and the nonparetic hands, while the other half only performed an active task on the paretic hand. One study combined fMRI with Transcranial Magnetic Stimulation (TMS) [18] and another combined fMRi, TMS, and Magnetoencephalography (MEG) [19]. TMS procedure was the same in the two studies; MEPs were recorded from the flexor pollicis brevis muscle from paretic and non-paretic hands by surface electromyography for amplitude and global transmission time [18, 19]. For MEG, somatosensory evoked magnetic fields (SEFs) were elicited by tactile stimulation of the paretic and non-paretic hands. Authors analyzed the tactile evoked magnetic field of the early SEF (amplitude and latency) [19]. Only one study evaluated the effects of reorganization trough voxel-based morphometry (VBM) analysis to determine gray matter change [14].

### 3.4. Findings

Details of the findings are reported in Table 3. To explore the effects of intervention on brain reorganization, neurofunctional techniques were used in 6/7 studies (fMRI in 6 studies, TMS in 2, MEG in 1), while a neurostructural technique (VBM) was used in 1/7 studies.

#### 3.4.1. Effects on the Hemisphere Contralateral to the PH (Affected or Most Affected Hemisphere)

The main neurofunctional effect upon the (most) affected hemisphere, observed after intervention, consisted of an enlargement of M1 or M1/S1 activation during active motor tasks of the paretic hand, demonstrated either at the single subject level [17] or at the group level [13, 15, 18, 19]. Less consistent findings were observed on fMRI during passive motor tasks of the paretic hand with enlargement of M1/S1 activation at the single subject level in one study [17], not confirmed at a group level in another study. Tasks performed by the non-paretic hand, both active and passive, did not seem to affect brain reorganization within the affected hemisphere. This general fMRI pattern was confirmed also when stratifying subjects according to motor reorganization (i.e., ipsilesional versus contralesional) [19]. In studies using TMS, a significant increase of M1-MEPs amplitude was observed, following the TMS stimulation of the affected hemisphere [18]. This finding was clearly not observed in subjects with contralesional reorganization of motor function, as no MEPs could be elicited in these subjects by stimulation of the affected hemisphere [19]. In the study using MEG, increased amplitude of the SEFs was observed in the affected hemisphere following tactile finger stimulation of the paretic hand, irrespective of the type of motor reorganization, while a reduction in SEFs latency was only observed in subjects with ipsilesional reorganization [19].

The only study exploring neurostructural changes, through VBM analysis [14], found an increased volume of M1/S1 in the affected hemisphere, together with an increased volume of the hippocampus.

#### 3.4.2. Effects on the Hemisphere Ipsilateral to the PH (Non-Affected or Least Affected Hemisphere)

No clear neurofunctional effects were observed upon the unaffected (or least affected) hemisphere. In a minority of cases, changes at the single subject level were observed on fMRI, at the level of M1/S1 or the Cerebellum [17]. On TMS, a small but significant decrease of M1-MEPs amplitude was observed, following TMS stimulation of the unaffected hemisphere, limited to those subjects with a contralesional reorganization [19]. No effects were observed using MEG, with the exception of a reduction of SEFs latency in the unaffected hemisphere following tactile finger stimulation of the non-paretic hand, limited to the subgroup of subjects with ipsilesional reorganization [19].

The only study exploring neurostructural changes, through VBM analysis, found an increased volume of M1 in the unaffected hemisphere, together with an increased volume of the hippocampus [14].

#### 3.4.3. Correlation of Brain Reorganization with Functional Improvement

The correlation between functional motor improvement in the upper limb and degree of neuroplastic changes was explored in 4/7 studies and significant correlations were found. In 3 studies CIMT was used and the training-related improvements were positively correlated with the extent of the area of activation [15], the laterality index [13] and volume increase at VBM [14]. In the study on VR, a correlation between motor function and fMRI signal during active motor tasks was found [16].

## 4. Discussion

Despite the high number of studies exploring the functional effects of neurorehabilitation in children with unilateral cerebral palsy, relatively little is known on the neurobiological underpinnings of such effects. The main common finding reported in the reviewed studies is the enlargement of the primary hand motor area contralateral to the paretic hand, following intervention. This was valid across different studies, both for CIMT [13–15, 17–19] and VR trainings [16], using various hand motor tasks such as finger tapping [15], hand opening/closing [16] and rubber ball pressing [17–19]. Contralateral primary motor and sensory cortex were the most frequently involved but increased activation could be also found in the supplementary motor area [18], the premotor cortex [17] and the cerebellum [16, 17]. More in general, the effect results into a shift in the laterality index due to the increased activity in the (most) affected hemisphere after therapy, not counterbalanced by a similar effect in the unaffected (or least affected) one.

The effect of training on hand passive motor task activation was less clear. Of the three papers exploring this question [17–19], one showed significant enlargements in about half of the tested subjects [17], the second one found no significant changes [18], while the third one found changes only in the subgroup of subjects with contralesional reorganization. The three studies however used different statistical approaches (single subject versus group analysis), making the three studies poorly comparable and potentially less conflicting.

In two of the six studies [14, 18], effects of training were explored with different means other than fMRI. Walther et al. [18] used TMS to determine the changes in corticospinal excitability following CIMT training, and recorded increased amplitude MEPs in the paretic hand from the contralateral primary motor cortex. No effect was observed for the non-paretic hand. Sterling et al. [14] explored the effects of training on a structural level by using VBM analysis. This is also the only paper with an actual control condition consisting of a same-length interval pretraining used to explore brain changes unrelated to intervention. It is of interest that while no changes were observed from baseline to pretreatment, a significant volume increase was observed at a group level posttreatment in the primary motor cortex bilaterally, in the contralateral primary sensory area and in both hippocampi.

Not surprisingly, a key factor influencing treatment-related brain neuroplasticity appeared to be the type of reorganization of the corticospinal tract (i.e., ipsilesional or contralesional). Type of reorganization was taken into account in 3/7 studies; these studies came from the same research group, with the most recent one [19] confirming and expanding the results of the previous two [17, 18]. Although the overall small figures do not allow for definite conclusions, there appears to be enough evidence supporting the existence of two types of treatment-related neuroplasticity with the main hallmark of an increase in M1 excitability in subjects with ipsilesional reorganization and of a decrease in M1 excitability in subjects with contralesional reorganization.

Positive effects of training on hand motor function, in the selected studies, were almost invariably reported, although the outcome measures used were very heterogeneous. When correlating functional improvements with the amount of plastic brain reorganization, significant results were generally observed after intervention, including enlarged area of M1S1 activation in fMRI, increased M1-MEPs amplitude from stimulation of the affected hemisphere, and increased M1S1 brain volumes on VBM. However, data are too scattered and heterogeneous to allow for definite conclusions on the possible correlations between neurobiological changes and functional improvements.

The main limitation of the findings of this review is related to the number and type of papers found in our systematic search. Studies included in this review consist of quasi-experimental or descriptive pre-post designs. Their level of evidence, based upon a modified Sackett score [23] adapted to include PEDro ratings, is between 2b for CIMT studies and 5 for VR. It is of great interest that none of the studies selected was a randomized controlled study. Although several RCTs have been performed comparing different trainings in children with unilateral cerebral palsy, some ethical problems might have hindered the possibility of testing control subjects with relatively invasive techniques such as TMS. Nevertheless, since MRI, MEG, and EEG techniques, when used without sedation, can be considered noninvasive, there is no obvious reason why RCTs have not yet been performed using these methods. Lack of RCTs might be more simply justified by this field of research being relatively new and this type of study design being more complex.

In summary, noninvasive rehabilitation strategies seem to produce measurable neuroplastic changes in sensory motor cortex associated with enhancement of motor skills in the affected limb. This conclusion is however largely restricted due to the strong limitations of the reviewed studies, the most relevant of which concerns their methodological characteristics. It is also important to underline that the selected studies only investigated the effects of two types of intervention, namely, CIMT and VR, making therefore our conclusions not applicable to other approaches. For the same reason, this review cannot provide any contribution to the definition of the type of intervention that should be recommended in children with U-CP. Well-designed experimental studies with larger sample sizes should be carried out to strengthen the generalizability of these preliminary findings. Moreover, for further studies it would be important to investigate the clinical outcomes according to the dimensions of ICF with the best measures created for children with hemiplegia considering psychometric properties. More researches, and in particular RCT studies, are needed to better understand the mechanism of brain plasticity in children with brain injury and to inform and fine-tune current or novel rehabilitation strategies in children with cerebral palsy.

## Supplementary Material

Table1s shows the results of the clinical assessments performed before and after the training. Results are shown either as single-subject scores or as group mean scores +/- the standard deviation. In one case only the significance of the pre/post-treatment increase is reported.

## Figures and Tables

**Table 1:** Population and study design.

Study	Design	Patients	M : F	Age	Diagnosis: congenital U-CP	Lesion/etiopathogenesis	Type of reorganization^§^
[13]	Case series	5*	n/a	7–13 ys	4R-CP, 1bilateralCP*	n/a	n/a
[14]	Case series	10	6 : 4	2.1–7.6 (3.3 ± 1.6)	8R-CP; 2L-CP	3L-FP; 4L-PV; 1R-F; 1L-FT; 1R-FP	n/a
[15]	Case series	10	4 : 6	7–14 ys (11 ± 2.5)	4L-CP; 6R-CP	3 malformative, 3 prenatal, 1 connatal, 2 early acquired, 1 n/a	n/a
[16]	Case series	3	2 : 1	13–15 ys	R-CP	2 patients: perinatal stroke, 1 patient: IVH	n/a
[17]	Case series	10	5 : 5	10–30 ys (median 14 ys)	6R-CP; 4L-CP	unilateral cortical-subcortical infarction in the MCA territory	7/10 ipsilesional 3/10 mixed
[18]	Case series	7	3 : 4	10–30 ys (median 16 ys)	5R-CP; 2L-CP	unilateral cortical-subcortical infarction in the MCA territory	ipsilesional
[19]	Case series divided into: contralesional, ipsilesional	16 9/16 7/16	8 : 8 5 : 4 3 : 4	10–31 ys 11–31 ys 10–30 ys	6L-CP, 10R-CP 4L-CP, 5R-CP 2L-CP, 5R-CP	unilateral cortical-subcortical infarction in the MCA territory	9/16 contralesional 7/16 ipsilesional

*A participant had bilateral involvement with right arm sparing; ^§^assessed by Transcranial Magnetic Stimulation (TMS). Abbreviations: M: male; F: female; ys: years; L: left; R: right; U-CP: unilateral cerebral palsy, IVH: intraventricular hemorrhage; MCA: middle cerebral artery; FP: frontoparietal; PV: periventricular; F: frontal; TP: temporal-parietal; CIMT: constraint-induced movement therapy; VR: virtual reality; NDT: neurodevelopmental treatment; OT: occupational therapy; n/a: not available.

**Table 2:** Characteristics of the UL intervention programs.

Study	Treatment	Duration	Frequency	Intensity per day	Environment	Activities	Restraining device or therapy system
[13]	Modify CIMT + OT	3 weeks	Weekly	n/a	In home	Bloorview Kids rehabilitation therapy manual	3 weeks continuous casting of the affected arm and hand
[14]	CIMT + intensive motor training	15 days*	Weekdays	5 hrs	N/a	Shaping technique	Less-affected arm is continuously restrained in a long arm cast
[15]	modify CIMT + NDT	2 weeks	Weekdays	4 hrs	Outpatient clinic, home, playgroup	Chosen collaboratively between child and therapists	Removable cast on nonaffected arm for 90% of the waking hrs included weekend
[16]	VR	2 months	Weekdays	30 min	In home	2 games: “sliders”, “chase away a butterfly”	5 DT5 Ultra Glove + Play Station 3 game console
[17]	CIMT + individual/peer group activities	12 days	Daily	n/a	Training camp	Individual (2 hrs) and peer group activities	Tailored glove fortified on palmer side and fingers (wearing time: 10 hrs/day)
[18]	CIMT + individual/peer group activities	12 days	Daily	n/a	Training camp	Individual (2 hrs) and peer group activities	Tailored glove fortified on palmer side and fingers (wearing time: 10 hrs/day)
[19]	CIMT + individual/peer group activities	12 days	Daily	10 hrs	Training camp	Individual (2 hrs) and peer group activities (8 hrs)	Tailored glove fortified on palmer side and fingers (wearing time: 10 hrs/day)

*On the last 2 days of treatment, the cast is removed and training is focused on bilateral activities. Abbreviations: CIMT: constraint-induced movement therapy; VR: virtual reality; NDT: neurodevelopmental treatment; OT: occupational therapy; n/a: not available; min: minutes; hrs: hours.

**Table 3:** Neuroimaging and neurophysiological outcome measures and results.

CIMT			
Functional magnetic resonance imaging	PH	N-PH	Notes

Active movements fMRI task			
Four-finger/wrist extension/flexion [13]	2/4 LI shift to contralateral hemisphere, 2/4 reduced LI (group stat; *n* = 4)	—	
Finger tapping [15]	6/7 (M1c) ↑ area of activation 2/7 (M1c) ↑ signal (1–3%)	—	3/10 were excluded due to artifacts (2/10) or claustrophobia. fMRI task was tested on 5 controls who showed M1c activation.
Rubber ball press [17]	1/3 (M1S1c + M1S1i + CBMi + PMC) ↑ area of activation	4/10 M1S1c ↑ area of activation 1/10 M1S1i ↑ area of activation	3/10 were excluded for PH task due to movement artifacts.
Rubber ball press [18]	(M1S1c + SMA) ↑ area of activation (group stat; *n* = 5)	No changes (group stat; *n* = 5)	2/7 were excluded for inability to perform the task.
Rubber ball press [19]	*Ipsilesional group*: (M1S1c + SMA) ↑ area of activation (group stat; *n* = 5)	No changes (group stat; *n* = 5)	2/7 were excluded for inability to perform the task.
	*Contralesional group* (M1S1c + CBMi/c) ↑ area of activation (M1i) ↓activation (group stat; *n* = 6)	No changes (group stat; *n* = 6)	3/9 were excluded for movement artefacts.

Passive movements			
Flexion/extension at the metacarpophalangeal of fingers II–V of the patient's hand [17]	4/8 (M1S1c) ↑ area of activation 1/8 (M1S1i) ↑ area of activation 2/8 (IHF) ↑ area of activation 1/8 (CMBi) ↑ area of activation 4/8 no changes observed	2/10 M1S1c ↑ area of activation 1/10 IHF ↑ area of activation	2/10 were excluded for PH task due to movement artifacts.
Flexion/extension at the metacarpophalangeal of fingers II–V of the patient's hand [18]	No changes (group stat; *n* = 7)	No changes (group stat; *n* = 7)	
Flexion/extension at the metacarpophalangeal of fingers II–V of the patient's hand [19]	*Ipsilesional group*: no changes (group stat; *n* = 7)	*Ipsilesional group*: no changes (group stat; *n* = 7)
	*Contralesional group* Parietal operculum_c_ + M2S2c ↓activation (group stat; *n* = 9)	*Contralesional group * M1S1c ↓activation (group stat; *n* = 9)

Voxel-based morphometry	Posttreatment—pretreatment	Pretreatment—baseline	Notes

VBM [14]	(M1S1c + M1i + Hippocampi) ↑ volume (group stat; *n* = 10)	No changes (group stat; *n* = 10)	

Transcranial magnetic stimulation	PH	N-PH	Notes

TMS [18]	(M1-MEPs) ↑ amplitude (group stat; *n* = 7)	No changes (group stat; *n* = 7)	
TMS [19] amplitude	*Ipsilesional group:* (M1-MEPs) ↑ amplitude (group stat; *n* = 7)	*Ipsilesional group:* No changes (group stat; *n* = 7)	
	*Contralesional group:* (M1-MEPs) ↓amplitude (group stat; *n* = 9)	*Contralesional group:* (M1-MEPs) ↓amplitude (group stat; *n* = 9)	
TMS [19] conduction time	*Ipsilesional group:* No changes (group stat; *n* = 7) *Contralesional group:* No changes (group stat; *n* = 9)	*Ipsilesional group*: No changes (group stat; *n* = 7) *Contralesional group:* No changes (group stat; *n* = 9)	

Magnetoencephalography	PH	N-PH	Notes

MEG [19] latency	*Ipsilesional group*: ↓ early-SEF latency (group stat; *n* = 7) *Contralesional group:* No changes in early-SEF latency (group stat; *n* = 8)	*Ipsilesional group:* ↓ early-SEF latency (group stat; *n* = 7) *Contralesional group:* No changes in early-SEF latency. (group stat; *n* = 8)	1/9 was excluded due to strong magnetic artefacts.
MEG [19] amplitude	*Ipsilesional group*: ↑ early-SEF amplitude (group stat; *n* = 7) *Contralesional group*: ↑ early SEF amplitude (group stat; *n* = 8)	*Ipsilesional group*: *≈* early-SEF amplitude (group stat; *n* = 7) *Contralesional group*: No changes SEF amplitude (group stat; *n* = 8)	1/9 was excluded due to strong magnetic artefacts.

VR			

Functional magnetic resonance imaging	PH	N-PH	Notes

Active movements fMRI task			
Hand open/close [16]	2/3 (M1c) ↑ area of activation 2/3 (CBM) ↑ area of activation	—	Training dose was variable in the 3 cases.

Abbreviations: PH: paretic hand; N-PH: nonparetic hand; fMRI: functional magnetic resonance imaging; VBM: voxel-based morphometry; TMS: transcranial magnetic stimulation, MEG: magnetoencephalography; M1: primary motor cortex; FP: frontoparietal; M1S1: primary sensory motor cortex, M2S2c secondary sensory motor cortex c/i: indicate contralateral/ipsilateral, CBM: cerebellum, IHF interhemispheric fissure (including cingulate motor area supplementary motor area), PMC: premotor cortex; LI: lateral index; LI is calculated [(contralateral − ipsilateral)/(contralateral + ipsilateral)]; SMA: supplementary motor area, MEPs: motor evoked potentials; SEF: somatosensory evoked potentials.
