# Supplementary material for: Brain Reorganization following Intervention in Children with Congenital Hemiplegia: A Systematic Review
Source: Neural Plast. 2013 Dec 3;2013:356275. doi: 10.1155/2013/356275 (PMC3866714; doi:10.1155/2013/356275)
Supplement: Supplementary file 1 — Table1s shows the results of the clinical assessments performed before and after the training. Results are shown either as single-subject scores or as group mean scores +/- the standard deviation. In one case only the significance of the pre/post-treatment increase is reported. [file 356275.f1.pdf]

Hand open/close [16]	2/3 (M1c) ↑ area of activation<br>2/3 (CBM) ↑ area of activation	-	Training dose was variable in the 3 cases

Abbreviations: PH: paretic hand; N-PH: non paretic hand; fMRI: functional magnetic resonance imaging; VBM: voxel-based morphometry; TMS: transcranial magnetic stimulation, MEG: magnetoencephalography; M1: primary motor cortex; FP: fronto-parietal; M1S1: primary-sensorymotor cortex, M2S2c secondary sensory-motor cortex c/i: indicate contralateral/ipsilateral, CBM: cerebellum, IHF interhemisferic fissure (including cingulate motor area supplementary motor area), PMC: premotor cortex; LI: lateral Index; LI is calculated [(contralateral-ipsilateral)/(contralateral+ipsilateral)]; SMA Supplementary motor area, MEPs: motor evoked potentials; SEF: somatosensory evoked potentials

Table 1s: Supplementary material: Clinical Outcome measure and results

Study	Clinical Outcome measure	n°	Results		
			Pre-treatment	Post-treatment	Follow-up
[13]<br>(n. sample= 5)	Body functions and structures				
	Grip strength mmHg:	1#	60	50.7	n/a
	Activity				
	QUEST<br>AHA	1#	23.1<br>25	24.7<br>27	n/a<br>n/a
	Participation				
	P-MAL AOU	1#	1.8	1.4	n/a
	Disregard index§ -12.9	1#			
	Body functions and structures				
	Grip strength mmHg	2#	30.7	39.3	n/a
	Activity				
	QUEST<br>AHA	2#	30.8<br>44	34.7<br>46	n/a<br>n/a
	Participation				
	P-MAL AOU	2#	1.6	2.3*	n/a
	Disregard index § -1.2	2#			
	BODY FUNCTIONS AND STRUCTURES				
	Grip strength mmHg	3#	102.7	129.7*	n/a
	ACTIVITY				
	QUEST<br>AHA	3#	69.2<br>68	68.7<br>76*	n/a<br>n/a
	Participation				
	P-MAL AOU	3#	0.6	4.7*	n/a
	Disregard index§ 57.2	3#			
	Body functions and structures				

	Grip strength mmHg:	4#	80		101.7*	n/a	
	**Activity**						
	QUEST<br>AHA	4#	72.6<br>62		79.0*<br>68*	n/a<br>n/a	
	**Participation**						
	P-MAL AOU	4#	0.53		3.3*	n/a	
	Disregard index[§] 62.6	4#					
	**Body functions and structures**						
	Grip strength mmHg:	5#	200		206.7	n/a	
	**Activity**						
	QUEST<br>AHA	5#	84.8<br>76		94.7*<br>80	n/a<br>n/a	
	**Participation**						
	P-MAL AOU	5#	3.8		3.7	n/a	
	Disregard index[§] 8.8	5#					
**[14]<br>(n. sample= 10)**	**Participation**		**Baseline**^	**Pre-treatment**^^	**Post-treatment**^^^		
	P-MAL-AUS-R	10	1.6± 0.8 points<br>14.9%	1.8 ± 0.8 points<br>No difference change respect to baseline	4.2 ± 0.7 points<br>50.8%*<br>Treatment Change: 2.4±0.6 (p< .001)	n/a	
**[15]<br>(n. sample= 10)<br><br>(Follow up at 3-6mths on 3 children)**	**Activity**					**3 mths**	**6 mths**
	Melbourne assessment	10	73.7		77.1* (p<.05)	66.9	75.7
	Kinematic analysis:	10					
	-peak velocity $cm\ s^{-1}$		41.1		48.2* (p <.05)	35	37.8
	-movement units		2.4		2.1	3.2	2.5
	-movements time reach in sec		1.3		1.2 ms	1.2	1.1
	-movements time grasp in sec		2.5		1.8 ms	2.3	2.3
	**Participation**						
	Parent's questionnaire	10	n/a		9/10 reported positive changes	n/a	n/a
**[16]<br>(n. sample= 3)**	**Body functions and structures**						
	Hand grip<br><br>ROM in thumb<br>ROM forefinger	3[a]<br><br>3<br>3	n/a		small improvement in all subjects<br><br>improved in all subjects<br>improved in 2 subjects	n/a<br><br>n/a<br>n/a	
	**Activity**						
	JHFT (7 items)	3	n/a		All 3 subjects reduce the execution time for 5 of 7 items	n/a	

	BOTMP	3		no significant changes	n/a
**[17]** **(n. sample= 10)**	**ACTIVITY**				
	WMFT time	10	1.86 ± 0.52 sec.	1.71 ± 0.70 sec. (p= 0.052)	n/a
	WMFT quality	10	3.45 ± 0.73	3.95 ± 0.71* (p= 0.005)	n/a
**[18]** **(n. sample= 7)** **Follow-up= 12 days**	**ACTIVITY**				**12 days**
	WMFT time	7	1.69 ± 0.33 sec	1.46 ± 0.41 (P= 0.018)	1.61 ± 0.38 (P= 0.446; P= 0.05)
	WMFT quality	7	3.73 ± 0.63	4.17 ± 0.70* (P= 0.016)	4.27 ± 0.79* (P= 0.018; P= 0.002)
	**PARTICIPATION**				**12 days**
	MAL	7	3.17 ± 0.53	3.32 ± 0.72 (P= 0.600)	4.01 ± 0.72* (P= 0.018; P= 0.013)
**[19]** **(n. sample= 16 divided in: ipsilesional and contralesional groups)**	**ACTIVITY IN IPSILESIONAL GROUP (N SAMPLE=7)**				
	WMFT time	7		significant increase (p=0.018)*	n/a
	WMFT quality	7		significant increase (p= 0.016)*	n/a
	**ACTIVITY IN CONTRALESIONAL GROUP (N SAMPLE= 9)**				
	WMFT time	9		no significant change (p= 0.051)	n/a
	WMFT quality	9		significant increase (p= 0.007)*	n/a

Baseline: 3 wks before receiving CI therapy; ^^Pretreatment: immediately before CI therapy; ^^^Post-treatment: immediately post CI therapy; [a] a subjects practiced only an half of trial due to quipment technical problems, * value reported as statistically significant. [§] disregard index = Quality of Upper Extremity Skills Test score – [Pediatric Motor Activity Log score/5 x 100 %]. bbreviations: n°: number of partecipants, JHFT: Jebsen Test, WMFT : Wolf Motor Function Test, time score and quality score , P-MAL-R: Pediatric Motor Activity Log-Revised; P-MAL-AUS: Pediatric Motor Activity Log Amount of Use Scale, P-MAL- QOM: Pediatric Motor Activity Log Quality of Movements; QUEST: quality upper extremity skills test; AHA: Assisting Hand Assessment, BOTMP: ruininks–Oseretsky Test of Motor Proficiency; n/a: not available.
